# Assessment of Cardiorespiratory Fitness without Exercise in Elderly Men with Chronic Cardiovascular and Metabolic Diseases

**DOI:** 10.1155/2012/518045

**Published:** 2011-12-11

**Authors:** Geraldo A. Maranhão Neto, Antonio P. de Leon, Vitor A. Lira, Paulo T. V. Farinatti

**Affiliations:** ^1^Department of Epidemiology, Institute of Social Medicine, Rio de Janeiro State University, 20550-900 Rio de Janeiro, RJ, Brazil; ^2^Laboratory of Physical Activity and Health Promotion, Institute of Physical Education and Sports, Rio de Janeiro State University, Rua São Francisco Xavier 524/Sala 8121F, Maracanã, 20550-900 Rio de Janeiro, RJ, Brazil; ^3^Division of Social Medicine, Karolinska Institute, Norrbacka, 171 76 Stockholm, Sweden; ^4^Robert M. Berne Cardiovascular Research Center, University of Virginia School of Medicine, 415 Lane Rd Charlottesville, VA 22908, USA; ^5^Physical Activity Sciences Graduate Program, Salgado de Oliveira University, Niterói, RJ, Brazil

## Abstract

Low cardiorespiratory (CRF) is associated with health problems in elderly people, especially cardiovascular and metabolic disease. However, physical limitations in this population frequently preclude the application of aerobic tests. We developed a model to estimate CRF without aerobic testing in older men with chronic cardiovascular and metabolic diseases. Subjects aged from 60 to 91 years were randomly assigned into validation (*n* = 67) and cross-validation (*n* = 29) groups. A hierarchical linear regression model included age, self-reported fitness, and handgrip strength normalized to body weight (*R* ^2^ = 0.79; SEE = 1.1 METs). The PRESS (predicted residual sum of squares) statistics revealed minimal shrinkage in relation to the original model and that predicted by the model and actual CRF correlated well in the cross-validation group (*r* = 0.85). The area under curve (AUC) values suggested a good accuracy of the model to detect disability in the validation (0.876, 95% CI: 0.793–0.959) and cross-validation groups (0.826, 95% CI: 0.677–0.975). Our findings suggest that CRF can be reliably estimated without exercise test in unhealthy elderly men.

## 1. Introduction

Cardiorespiratory fitness (CRF) maintenance is important for functional independence and physical capacity throughout aging [1, 2]. Substantial declines in the ability to tolerate physical exertion generally predict mobility problems and cardiovascular morbidity and mortality, particularly in the sedentary elderly [3, 4]. Despite the importance of CRF assessment, very low functional capacity and frailty may hinder the use of exercise tests in this population [5, 6]. In this context, nonexercise prediction models become practical alternatives to estimate CRF [7] and may have important applications both in clinical and epidemiological settings. These models are developed by means of regression-based equations that usually include variables of simple and fast assessment, such as anthropometric measures, demographic characteristics, and daily habits [8]. 

Recently, Mailey et al. [7] cross-validated an equation developed primarily in middle-aged adults by Jurca et al. [9] and suggested that nonexercise models could be used to estimate the CRF of older adults. The sample studied was mainly composed by healthy old women (~60%). However, the prevalence of chronic diseases such as cardiovascular disease and diabetes increases dramatically with age [10], and is associated to lower physical capacity, inactivity, and limitations in the ability to exercise [2]. It would be therefore important to take into account that elderly populations are not always healthy and free of cardiovascular and metabolic diseases, which on the contrary, are common in the later life. Notwithstanding to date nonexercise models to assess the CRF in elderly subjects with chronic diseases have not been proposed. Hence the present study aimed to develop a CRF nonexercise prediction linear model of cardiorespiratory fitness and test its validity in elderly men presenting chronic cardiovascular and metabolic diseases.

## 2. Materials and Methods

### 2.1. Subjects

The sample consisted initially of 108 subjects admitted to the Elderly Care Center of the Open University of the Third Age (UNATI) of the Rio de Janeiro State University (UERJ). These subjects went through clinical exams in order to detail their medical history and completed a brief questionnaire providing demographic information, which was used to determine whether they could perform maximal aerobic exercise testing. 

The inclusion criteria were asymptomatic patients, stable disease, and no abnormalities in rest electrocardiogram for at least six months. Tests interrupted due to clinical reasons were not considered as maximal and therefore have been excluded from the study. Twelve participants did not complete the exercise testing (five were not allowed by the physicians to perform the test, four asked to stop before achieving maximal effort, and three exhibited high blood pressure).

From the initial sample, a total of 96 subjects remained in the study (60–91 years), being randomly assigned into two groups: validation (70%) and cross-validation (30%). The command “sample 70” from STATA statistical package version 10.1 (Stata, College Station, TX) was used with this purpose. The validation group was used to develop the statistical model while the cross-validation group was used to confirm the generalization potential of the obtained model. 

The experimental protocol was approved by institutional Ethical Committee and participants provided a written informed consent for the use of their data for research prior to the commencement of the study, as stated in the Declaration of Helsinki.

### 2.2. Procedures

Individual data on anthropometry, self-reported physical fitness, and physiological measurements were assessed. Anthropometric measurements included weight and height [11] from which the body mass index (BMI) was calculated. The physical activity history was assessed by the Self-Reported Physical Activity Index (SRPA) [9]. The subjects were asked to choose one of five activity categories that best described their usual pattern of daily physical activities. Self-rated fitness was evaluated by means of the Rating of Perceived Capacity scale (RPC), a 1-20-scale previously adapted and translated to the Brazilian Portuguese language [12], in which the subject chooses the most strenuous activity that can be sustained for at least 30 min [13]. The RPC score is expressed in METs and the listed activities include walking, jogging, running, and cycling at different paces. Instead of asking of the physical activity history, the scale focuses on which activity the subject is able to perform. 

The blood pressure at rest and during exercise was measured by auscultation with a sphygmomanometer WelchAlln (Tycos, Arden, MN, USA). A 12-lead electrocardiogram was used to assess the resting heart rate (RHR) and maximal heart rate (Micromed, Brasilia, DF, Brazil). Handgrip strength (HG) was measured with subjects keeping their shoulder adducted and neutrally rotated, with the arm fully extended and being encouraged to exert maximal grip force on a Lafayette dynamometer 78010 (Lafayette, IN, USA). The highest value in kilograms (kg) was determined after four trials in the dominant and nondominant hands and the relative handgrip strength (i.e., handgrip strength normalized to body weight and represented as the ratio handgrip/weight) was then used for further analyses.

All subjects performed a clinically supervised maximal exercise test in an electromagnetically braked cycloergometer (Cateye EC-1600, Osaka, Japan) using an individualized ramp protocol. Subjects were submitted to a familiarization trial to get used to the cycloergometer and mouthpiece on the day prior to the exercise test. The Wasserman et al. [14] prediction equation was used to estimate the test incremental workload in watts in order to achieve maximal exertion in approximately 10 minutes. The metabolic analyzer VO2000 (Medical Graphics, St. Paul, MN, USA) was used for gas exchange measures, using a medium flow pneumotachometer (10–120 L·min^−1^). 

Peak oxygen uptake (VO_2  peak_) was determined as the maximal oxygen uptake at the point of test termination due to volitional exhaustion. A MET value of 3.5 mL·kg·min was considered for further calculations. The Borg CR-10 perceived exertion scale was used to estimate the degree of exertion [15] and standard clinical criteria for terminating exercise testing have been applied [16]. Before each test, the equipment was calibrated as recommended by the manufacturer, using standard reference gases. The test was considered maximal when at least two of the following criteria were observed: (a) respiratory exchange ratio (RER) > 1.1, (b) VO_2_ plateau despite an increase in workload (increase <2.0 mL·kg^−1^·min^−1^ between the last two loads), and (c) maximum volitional exhaustion.

### 2.3. Statistical Analyses

Data normality was confirmed by univariate analysis. Differences between the validation and cross-validation groups were analyzed by *t*-test Student's and chi-square tests. Age, relative strength index (handgrip strength/body weight), an anthropometric measure (BMI), self-related fitness (RPC scale), self-related physical activity (SRPA index), and resting heart rate (RHR) were entered, respectively, into the first, second, third, fourth, fifth, and sixth blocks of the hierarchical linear regression procedure to develop a model to predict MET_peak_ (i.e., the number of METs achieved at VO_2  peak_). 

The coefficient of determination (*R* ^2^) and the standard error of estimate (SEE) were calculated and the prediction equation generated was then cross-validated using the PRESS method (predicted residual sum of squares), a statistical jack-knife procedure that consists of refitting the proposed model many times, leaving each observation out of the model fit in turn, so as to predict and then calculate the residual for that observation [17]. The second series of cross-validation analyses based on the independent sample was performed using the Pearson correlation between the MET_peak_ predicted by the model and the MET_peak_ measured, as well as the difference between the CRF estimated by the prediction model and measured CRF (defined in the maximal exercise testing) was analyzed by the *t*-test. A Bland-Altman analysis of measurement differences plotted versus mean values was used to assess the degree of agreement [18]. 

In order to evaluate the accuracy of the model in screening individuals that present very low cardiorespiratory fitness, and therefore are at higher risk for mortality and morbidity, a disability index was used to dichotomize CRF. Basically, the CRF level below which successful independent living is significantly compromised (i.e., 5 METs) [19], was used as a cutoff to calculate the model sensitivity, specificity, and the area under the receiver operation curve (AUC). A 95% confidence interval (95%) was also calculated. The AUC is a measure of diagnostic power of the model and describes the probability that the model will correctly identify subjects with low CRF. The predicted and measured CRF categories from the validation and cross-validation groups were cross-tabulated to detect classification accuracy.

## 3. Results

Table 1 presents the descriptive statistics, as well as values for cardiorespiratory variables at rest and in response to maximal exercise for subjects in both validation and cross-validation groups. These groups did not significantly differ in any of the variables studied (*P* > 0.05). All subjects attended at least two of the criteria adopted to consider the cardiopulmonary exercise testing as maximal: respiratory exchange ratio (RER) > 1.1 (87% of the sample), VO_2_ plateau despite an increase in workload (75%), and (c) maximum volitional exhaustion (100%). 

Three levels of physical activity were detected by the SRPA index: inactivity or little activity other than usual daily activities (58% of the whole sample), participation in physical activities requiring low levels of exertion that result in slight increases in breathing and heart rate for at least 10 minutes at a time (38%), and participation in aerobic exercises such as brisk walking, jogging or running, cycling, swimming, or vigorous sports at a comfortable pace or other activities requiring similar levels of exertion for 20 to 60 minutes per week (4%).

Only three of four variables were significantly associated (*P* < 0.01) with CRF after the linear regression procedure (Table 2). Age, relative handgrip strength, and RPC explained 44%, 22%, and 13% of CRF variance, respectively. The BMI (*R* ^2^ = 0.003, *P* = 0.44), SRPA index (*R* ^2^ = 0.003, *P* = 0.37), and RHR (*R* ^2^ = 0.001, *P* = 0.53) were not significant predictors. Based on the *R* ^2^ value, the obtained model explained 79% of the variability in VO_2  peak_. The low shrinkage of adjusted *R* ^2^ and the *R* ^2^-PRESS conferred a good generalization to the model. The SEE_p_ was essentially equal to the corresponding SEE value. The following prediction equation was generated from the coefficients presented in Table 2: MET_peak_ = 6.095 − 0.096 (Age) + 8.84 (Handgrip strength/Weight) + 0.67 (RPC).

**Figure 1:**
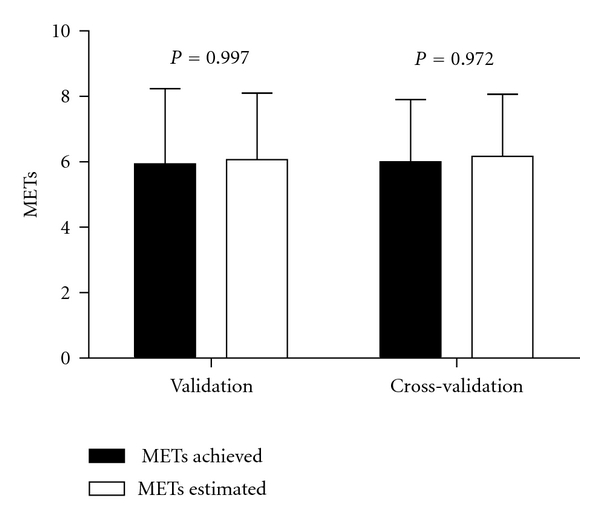
Predicted and actual CRF (MET_peak_; mean ± SD) in both validation and cross-validation groups.

**Figure 2:**
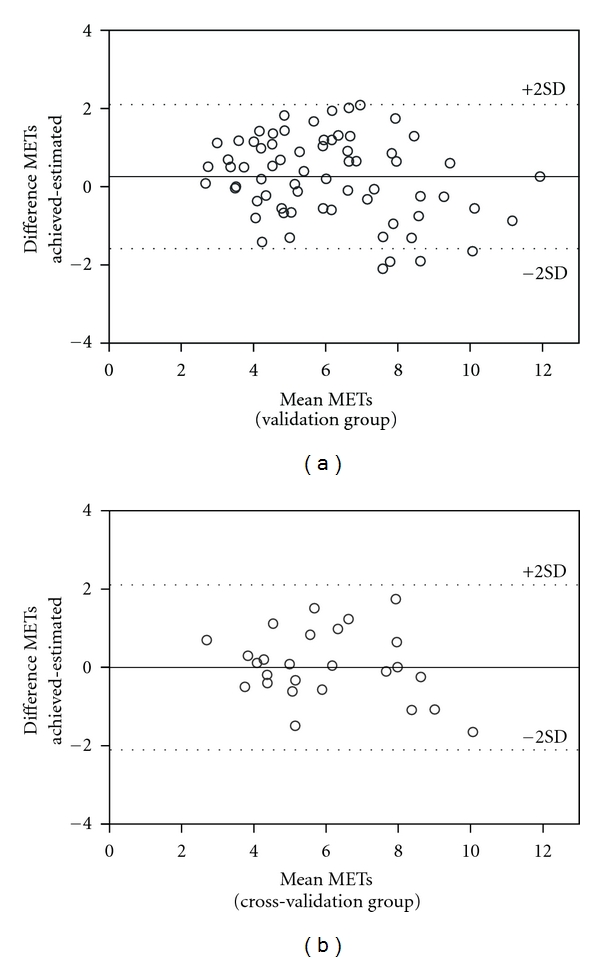
Bland Altman plot for predicted and actual CRF in both validation and cross-validation groups.

Once the statistical model was established it was then further tested in an independent sample for cross-validation. There were no significant differences between predicted CRF mean and actual CRF mean in both validation and cross-validation groups (Figure 1). The Pearson correlation coefficient between predicted and actual CRF in the cross-validation group was 0.85 (*P* < 0.01), ratifying that the model was appropriate to estimate CRF in elderly subjects with cardiovascular and metabolic diseases. The Bland Altman analysis (Figure 2) showed that few values fell outside the ranges established by the ±2 SDs, which suggests a good agreement between the values estimated by the statistical model and the actual MET values. 

Table 3 exhibits the model accuracy from the validation and cross-validation groups after diagnostic tests. The results demonstrated that the model presents very good sensitivity and specificity. The model was almost equally efficient in identifying subjects with CRF ≤ 5 METs and with CRF > 5 METs. The AUC further confirmed the high accuracy of the model in screening subjects presenting CRF either below or above 5 METs.

## 4. Discussion

 The negative effect of aging on CRF as well as the use of self-reported fitness are both well documented in the literature and are frequently included in prediction models [8, 20]. Although not previously used in other models, the relative handgrip strength was a very significant predictor of CRF in the present study. One could argue that the inclusion of handgrip strength may limit the widespread applicability of this equation, given that it requires equipment that may not be routinely used in epidemiological studies. In fact, the handgrip dynamometry has been a very important tool to assess the functional status of elderly samples, and the inclusion of this measurement in epidemiological settings should be reconsidered. The handgrip strength test is very simple and inexpensive, and has been previously related to mortality, mobility, functional capacity, and correlated with walking speed and overall strength, which are variables strongly related to the functional independence and health in older persons [1, 21, 22]. 

Despite of the fact that 79% (*R* ^2^) of variance in the prediction of CRF was explained by the obtained statistical model, the SEE (Table 2) suggests that more precise methods as maximal tests are recommended if the exactly CRF value is needed. On the other hand, the AUC values (Table 3) suggested that the model has good accuracy to stratify elderly men with very low (i.e., CRF ≤ 5 METs) and higher cardiorespiratory capacity (i.e., CRF > 5 METs). In other words, the model was capable to identify elder subjects whose functional and exercise capacity are compromised. Based on this finding, the proposed model can be useful for population-based investigations or epidemiological studies, especially those searching for associations between CRF and other physical and mental health outcomes, such as cognitive function and wellbeing. In clinical studies our model could be used to screen elderly individuals before maximal exercise testing. 

Mailey et al. [7] tested the validity of the nonexercise model proposed by Jurca et al. [9] in old adults, and reported a multiple *R* of 0.72 (*P* < 0.001) for the regression in a sample composed mainly of old women, which were somewhat more physically active than our subjects. Our model was developed in a sample of elderly men with cardiovascular and metabolic diseases and produced a multiple *R* of 0.89 (*P* < 0.001). It is also worthy to note that metabolic and cardiovascular diseases in the elderly are highly prevalent [10], reinforcing the relevance of the present model for this population. 

Although comparisons between the two models are difficult because they were developed in different populations, we have included variables from Mailey's model in our regression model, and they were not significant. We have also compared the CRF estimation accuracy of the models, and the results were favorable to the present equation (Mailey's model in the validation group: *R* ^2^ = 0.47; EPE = 1.7 METs, and in the cross-validation group: *R* ^2^ = 0.33; EPE = 1.6 METs). 

The adoption of a cycloergometer maximal exercise testing protocol to assess the MET_peak_ must be justified. Although treadmill tests are known to engage larger muscle mass and therefore may elicit higher peak VO_2_ [16, 23], some authors have proposed that cycloergometer tests would be more appropriate to assess the cardiorespiratory fitness in older subjects, mainly for safety reasons. For instance, it has been suggested that high-intensity treadmill exercise should be avoided in older subjects with balance restrictions or joint problems [24]. Moreover, the poor mechanical efficiency while running seems to reduce the performance of older compared to younger subjects during treadmill exercise [25], which would very likely limit the peak VO_2_ in maximal cardiopulmonary tests. Such limitation has been considered by previous research that adopted cycloergometer protocols to assess the cardiorespiratory fitness in older populations [4, 26].

The main finding of this study was that CRF of elderly men with cardiovascular and metabolic diseases and low physical capacity can be classified without aerobic tests using a combination of information on the subject's daily activity levels, relative handgrip strength, and a self-report of physical fitness level. Maximal aerobic tests have a higher cost, demand familiarization with ergometers, and are frequently difficult to perform in old adults due to poor balance and coordination, gait problems, and fear of exercising [27]. It is also worthy to mention that the accuracy of some submaximal exercise testing models to estimate CRF [28, 29] could be comparable to the accuracy of the present prediction model. It also represents an alternative to some walking tests because it does not require encouragement, which can be a source of disparity across trials, and does not require fatigue and dyspnea measurements. 

This study has some limitations, namely, the relatively small sample size, the prediction model restricted to unhealthy men. It is worthy to mention that since running tests may potentially produce higher MET_peak_ values, the reproducibility of our findings in treadmill exercise testing should also be addressed, despite the fact that cycloergometer tests are frequently indicated due to safety and mechanical efficiency reasons. Another issue refers to whether the model is capable to show changes in fitness. Probably, changes in age, handgrip strength, and self-related fitness would influence the functional capacity and the METs levels, but only longitudinal studies could confirm this possibility. Additionally, the main objective of the present study was similar to other studies that developed nonexercise models, which were not conceived to detect slight longitudinal variations. In brief, the model aims to classify and compare individuals within a given population, and does not intend to replace cardiopulmonary exercise testing to precisely assess the CRF. Therefore, it is possible that small changes in CRF due to training cannot be detected by our equation.

## 5. Conclusion

CRF level is an important indicator of morbidity and mortality in elderly men. However, the CRF assessment is usually limited due the mobility issues and the risk of cardiovascular events especially in older people with chronic conditions. The present study presented an accurate fitness prediction model for elderly men with cardiovascular and metabolic diseases. The model provides a very fast and safe assessment of fitness, without any chance for cardiovascular events during assessment, which could be very feasible in many healthcare settings to estimate CRF and stratify elderly subjects accordingly, and very attractive for epidemiological studies.

## Figures and Tables

**Table 1:** Subjects' characteristics (validation and cross-validation groups).

	Validation (*n* = 67)	Cross-validation (*n* = 29)
Age (years)	69.1 ± 7.4	68.7 ± 6.6
Height (cm)	172.4 ± 6.8	172.9 ± 6.1
Weight (kg)	82.7 ± 16.0	83.9 ± 11.7
BMI (kg/m^2^)	27.7 ± 4.6	28.3 ± 4.1
Handgrip/weight	0.39 ± 0.09	0.40 ± 0.08
RPC*	4.8 ± 1.5	4.7 ± 1.5
Resting heart rate (bpm)	70.3 ± 15.2	69.7 ± 13.0
VO_2 peak_ (mL/kg/min)	20.8 ± 8.0	20.9 ± 6.8
MET_peak_	5.9 ± 2.3	6.0 ± 1.9
Peak heart rate (bpm)	131.7 ± 27.3	134.1 ± 30.3
Peak watts	106.9 ± 46.1	112.5 ± 42.4

Clinical history (%)		
Cardiovascular disease	39	38
Obesity	22	27
Smoking	7	2
Hypertension	63	52
Diabetes	10	7
Cholesterol level >220	21	21
History of myocardial infarction	15	17
Arrhythmia	7	14
History of revascularization	24	24
History of coronary angioplasty	13	10
Musculoskeletal problems	6	4
*β*-blocker usage	36	35

*Rating of perceived capacity scale.

**Table 2:** Prediction model after stepwise multiple regression (validation group).

Predictor variables								
Constant	Age	Handgrip strength/weight	RPC	*R* ^2^	SEE	*R* ^2^ adjusted	*R* ^2^_p_	SEE_P_
6.095 (1.851)	−0.096** (0.020)	8.840** (1.601)	0.670** (0.104)	0.79	1.1	0.78	0.76	1.1

**P < 0.001; RPC: rating of perceived capacity; SEE: standard error of estimate (values in METs). Numbers within parentheses are the standard regression coefficients.

**Table 3:** Diagnostic accuracy to detect low cardiorespiratory fitness (CRF ≤ 5 METs).

Group	Sensitivity	Specificity	AUC
Validation	0.852 (0.663–0.958)	0.90 (0.763–0.972)	0.876 (0.793–0.959)
Cross-validation	0.818 (0.482–0.977)	0.833 (0.586–0.964)	0.826 (0.677–0.975)

95% CI in parentheses; AUC: area under the curve.
